# Endoscopic ossiculoplasty: audiological and surgical outcomes from a multicenter experience with 292 cases

**DOI:** 10.1007/s00405-025-09473-y

**Published:** 2025-05-29

**Authors:** Raffael Fink, Giulia Molinari, Arianna Burato, Daniel Stricker, Nicola Bisi, Daniele Marchioni, Livio Presutti, Ignacio Javier Fernandez, Lukas Anschuetz

**Affiliations:** 1 Department of Otorhinolaryngology, Head and Neck Surgery, Bern University Hospital, Inselspital, University of Bern, Bern, Switzerland; 2 Department of Otolaryngology, Head and Neck Surgery, IRCCS Azienda Ospedaliero-Universitaria di Bologna, Bologna, Italy; 3 Alma Mater Studiorum-Università di Bologna, Bologna, Italy; 4 Institute of Medical Education, University of Bern, Bern, Switzerland; 5 Department of Otorhinolaryngology, Head and Neck Surgery, University Hospital of Modena, Modena, Italy; 6 Department of Otorhinolaryngology, Head and Neck Surgery, Santa Maria delle Croci Hospital Ravenna, Ravenna, Italy; 7 Department of Otorhinolaryngology, Head and Neck Surgery, CHUV, University of Lausanne, Lausanne, Switzerland; 8 The Sense Innovation and Research Center, Lausanne and Sion, Lausanne, Switzerland

**Keywords:** Endoscopic ossiculoplasty, Audiological outcomes, Ossicular replacement prostheses, Air-bone gap, Ear surgery, Mastoid preservation

## Abstract

**Purpose:**

To evaluate surgical and audiological outcomes of different endoscopic ossiculoplasty (endo-OPL) techniques and prosthesis materials.

**Methods:**

In this multicenter retrospective study, 292 patients underwent endo-OPL for various ossicular chain pathologies at three university hospitals between 2017 and 2023. We analyzed pre- and postoperative hearing metrics, perforation characteristics, surgical procedures, prosthesis types, and complication rates.

**Results:**

The cohort showed significant audiological improvement, with the mean preoperative air-bone gap (ABG) reducing from 26.88 dB (SD ± 12.73) to 19.94 dB (SD ± 10.90) at the last follow-up (FU; mean 20.7 months ± 15.53) (*p* = 0.001), and a graft success rate of 94.2%. Significant postoperative ABG improvements were observed with bony partial ossicular replacement prostheses (PORP) (*p* < 0.001), titanium total ossicular replacement prostheses (TORP) (*p* = 0.008), and semi-synthetic TORP (*p* = 0.016). Both cement PORP and bony PORP groups showed consistent ABG improvements over two FU visits. Conversely, titanium based PORP and TORP demonstrated initial improvements but later showed deterioration. Prosthesis extrusion and dislocation rates were 8.4% and 4.2% for titanium PORP, and 0% and 5.2% for titanium TORP, respectively, at the last FU. Endo-OPL combined with additional mastoidectomy (*n* = 59) did not yield significant ABG improvements.

**Conclusion:**

This multicenter study supports the efficacy of endo-OPL as a valuable technique to improve hearing outcomes in patients with conductive or mixed hearing loss. It highlights the superior performance of specific prosthesis materials and demonstrates that endo-OPL without additional mastoidectomy provides better hearing results, emphasizing the benefits of mastoid preservation in exclusive endoscopic procedures.

## Introduction

Ossiculoplasty (OPL) is a surgical procedure performed to restore the sound-transmitting mechanism of the middle ear. The objectives of OPL are to ensure mobility across all elements of the ossicular chain and to re-establish continuity between the tympanic membrane and the oval window. The earliest attempts at reconstructing the ossicular chain to improve hearing date back to the 19th century. Kessel first described myringostapediopexy in 1885, and Matte further pioneered OPL in 1901 [1, 2]. Since then, it has become evident that the success of OPL depends on several factors, including the extent of middle ear pathology, the reconstruction technique and prosthesis properties. Larger defects with absence of stapes suprastructure or chronic infection, are often associated with poorer results due to the extensive mucosal damage, which could hinder healing and postoperative middle ear ventilation [3–6].

The literature reports successful air-bone gap closure to within 20 dB in approximately 60–80% of cases, with prosthesis stability and absence of extrusion being critical determinants of success. Reconstruction options depend on the size of the defect in the long process of the incus and/or stapes, with partial ossicular replacement prostheses (PORPs) generally yielding better hearing outcomes than total ossicular replacement prostheses (TORPs) in the same procedure. According to a recent review by Beckmann et al., results from bridging tympanoplasty type II are superior to the results after type III tympanoplasty [7, 8]. 

Regarding materials, cartilage and incus interposition are considered the most favorable autologous options due to their biocompatibility and long-term stability [9, 10]. Among alloplastic materials available for ossicular reconstruction, titanium is the most widely used due to its consistent results in improving hearing, as supported by recent systematic reviews and meta-analyses [11]. However, despite the growing body of research, no material has yet been conclusively proven to provide statistically superior hearing outcomes [12]. 

Exclusive endoscopic ossiculoplasty (endo-OPL) represents a relatively new advancement in ear surgery, offering advantages such as a panoramic view of the middle ear and close visualization of critical areas, including the oval window region, the stapes suprastructure, and the footplate– factors that facilitates assessment of defects and prosthesis positioning [13, 14]. Studies comparing endoscopic versus microscopic OPL have demonstrated several advantages of the endoscopic approach, such as shorter operative times (up to 25% reduction in some cases), the avoidance of retroauricolar incision and canalplasty, and reduced morbidity [15, 16]. Additionally, a 40% reduction in the risk of postoperative complications in the endoscopic group (ear pain, numbness, and ear discharge) has been reported [17]. Eventually, endo-OPL seems to provide a smoother learning curve for trainees, with 70% of surgeons reporting faster skill acquisition and greater ease in procedure performance endoscopically [18–20]. 

Despite these advantages, evidence on short- and long-term outcomes of endo-OPL, particularly from large case series, remains limited. There is a`lso a need for more detailed analysis regarding the use of different partial or total prostheses, and various reconstruction materials. This study aims to close this gap by comparing the surgical and audiological outcomes from different endo-OPL techniques and materials, drawing from a multicenter experience.

## Methods

### Study design and participants

This retrospective study was conducted on patients with conductive or mixed hearing loss who underwent OPL via an endoscopic transcanal approach at the Departments of Otorhinolaryngology, Head and Neck Surgery at Inselspital, Bern, Switzerland, at Bologna University Hospital, Italy and Modena University Hospital, Italy between 2017 and 2023. In cholesteatoma cases requiring mastoidectomy due to disease extension beyond the lateral semicircular canal into the mastoid, OPL was conducted endoscopically, according to Cohen Class 2b classification [21]. Patients classified as Cohen Class 2a or lower, or those lacking follow-up (FU) data, were excluded from the analysis. All procedures conformed to the ethical standards established by the national research committees and the World Medical Association Declaration of Helsinki (2002). Ethical approval for this study was obtained from the institutional and regional review boards (Kantonale Ethikkomission, KEK-BE 2019-00555 and CE-AVEN: 0000872/22).

### Data collection

Data on pre- and postoperative hearing function, surgical procedure (including disease extent, ossicular chain status and type of OPL) were retrospectively collected from patient records and FU visits. Pre- and postoperative audiological assessments were compared, with a postoperative assessment conducted at a minimum of three months and, if available, at the last FU visit. Assessments included otoendoscopy and pure-tone audiometry, with bone conduction (BC) and air conduction (AC) pure-tone averages (PTA) calculated from thresholds at 0.25, 0.5, 1, 2, and 4 kHz frequencies. The mean air–bone gap (ABG) was determined as the difference between AC-PTA and BC-PTA. ABG improvement was calculated as the difference between mean preoperative ABG and mean postoperative ABG values, with a positive value indicating improvement.

### Surgical technique

A transcanal endoscopic approach was used in all patients, employing a 3-mm diameter, 14-cm length, 0° rigid endoscope under general anesthesia. Xenon light intensity was set at 50% of maximum power, in line with standard practice for endoscopic ear surgery (EES) at all centers. For patients affected by cholesteatoma, ossicular reconstruction was performed concurrently with disease eradication in all patients from Bern University Hospital and for 146 out of 149 patients (98%) from Bologna and Modena University Hospitals. Three out of 149 (2%) received endo-OPL during second-look procedures, all of whom were pediatric patients.

The choice between PORP or TORP was based on the intraoperative presence or absence of the stapes suprastructure, respectively. Prosthesis selection depended on the availability of suitable autografts and the surgeons’ experience. For heterologous reconstructions, all synthetic prosthesis were titanium-based and were covered with cartilage on their lateral surface to reduce extrusion risk. Prosthesis length was tailored according to intraoperative measurements and all prostheses were secured using resorbable pledgets. After tympano-meatal flap repositioning, the external auditory canal was packed with resorbable material, with or without silk dressing, based on the surgeon’s preference.

### Statistical analysis

All data were exported to the Statistical Package for Social Sciences (SPSS), version 29 (IBM Corp., Armonk, NY). Descriptive and inferential statistical analyses were done, depending on the variables being tested. Significance tests were completed to examine the relationships between variables. A split plot analysis of variance (ANOVA) with time of measurement (preoperative vs. last FU) as within subjects’ factor and material used for partial or total reconstruction as between subjects’ factor was done to gain insight of the improvement of the ABG (dependent variable).

In a post-hoc analysis separate paired t-tests were used for each material to check significant improvement of the ABG from preoperative to the last FU in detail.

In a second split plot ANOVA the progress of ABG (dependent variable) over time was analyzed in more detail separately for partial and total reconstruction and the material chosen. Time of measurement (preoperative, 1st FU and last FU) served as within subjects’ factor and material as between subjects’ factor. A trend analysis over time was applied for materials where appropriate to check if the ABG improvement remained stable from the first to the last FU. Finally, three paired samples t-tests were conducted to reveal effects of surgical technique over time (preoperative and last FU) on the ABG (dependent variable). T-tests were performed separately for the three surgical techniques (endo-OPL, endo-OPL with canal wall down (CWD) and endo-OPL with canal wall up (CWU)).

P-values less than 0.05 were considered statistically significant. Partial eta-squared ($\:{\eta\:}_{p}^{2}$) is reported as a mean of effect size where appropriate. In all ANOVAs all tests of within subjects’ effects were corrected after Huynh-Feldt if the sphericity assumption was violated. Multiple post-hoc comparisons of mean ABG were Bonferroni corrected.

## Results

### Cohort characteristics

A total of 292 patients were included in the study, with a mean age of 37.2 years (SD ± 19.4) at the time of surgery. The cohort consisted of 41% females and 59% males, with an even distribution of left-sided (50%) and right-sided (50%) ear surgeries. Most patients had a history of chronic otitis media (COM) with cholesteatoma (70%), followed by patients affected by COM without cholesteatoma (23%). The remaining 7% underwent surgery for ossicular malformation or atelectasis of the tympanic membrane. Additionally, 31% of patients had undergone prior surgery on the affected ear.

Among all procedures performed 232 (80%) were classified as Cohen class 3. Table 1 provides a comprehensive summary of patient demographics and surgical details, including PORP and TORP techniques employed.

**Table 1:** Demographics and surgical data

	*N* = 292	%
**Female**	121	41%
**Left ear**	147	50%
**Revision**	89	31%
**Underlying disease** COM with cholesteatoma COM without cholesteatoma Other	203 68 21	70% 23% 7%
**PORP Total** Bony Direct cartilage Titanium Double cartilage block Cement	**221** 136 29 24 23 9	**76%** 47% 10% 8% 8% 3%
**TORP Total** Titanium Bony Semi-synthetic	**71** 39 18 14	**24%** 13% 6% 5%
**Mastoidectomy** CWU CWD	**59** 44 15	**20%** 15% 5%

Abbreviations: COM = chronic otitis media; CWD = canal wall down.; CWU = canal wall up; PORP = partial ossicular replacement prosthesis; TORP = total ossicular replacement prosthesis

### Audiological outcomes of endo-OPL

The mean FU was 20.7 months (± 15.53). Overall, the preoperative mean ABG significantly improved from 26.88 dB to 19.94 dB at last FU (*p* = 0.001). Given the multiple comparisons conducted, all p-values were Bonferroni-corrected to adjust for potential Type I errors. A statistically significant postoperative ABG improvement was observed for bony PORP (*p* < 0.001), titanium TORP (*p* = 0.008), and semi-synthetic TORP (*p* = 0.016). Although other PORP reconstructions demonstrated a reduction in ABG, it did not reach statistical significance, mainly due to the lesser amount of observations in these categories. Table 2 provides detailed insights into the specific techniques utilized and their respective pre- and postoperative ABG values.

**Table 2:** Audiological outcomes according to the reconstruction (TORP vs. PORP) and material used

Technique	Material	*n*	Pre-operative ABG Mean (± SD)	Post-operative ABG Mean (± SD)	*p* value
**PORP**	Bony (Incus, Malleus, Cortical bone)	136	24.15 (± 12.88)	16.42 (± 11.02)	< 0.001*
	Titanium	24	27.25 (± 9.77)	22.33 (± 13.13)	n.s.
	Double cartilage block	23	26.99 (± 12.82)	22.02 (± 10.43)	n.s.
	Direct cartilage	29	24.95 (± 12.54)	20.17 (± 11.22)	n.s.
	Cement	9	26.33 (± 15.84)	15.28 (± 5.93)	n.s.
**TORP**	Bony (Incus or Cortical bone)	18	27.68 (± 11.46)	27.44 (± 13.89)	n.s.
	Titanium	39	32.26 (± 10.18)	24.44 (± 10.58)	0.008*
	Semi-synthetic	14	40.90 (± 10.07)	27.05 (± 13.92)	0.016*
	**Total**	292	26.88 (± 12.73)	19.94 (± 10.90)	0.001*

Abbreviations: ABG = Air bone gap; PORP = partial ossicular replacement prosthesis; SD = standard deviation; SD = standard deviation; TORP = total ossicular replacement prosthesis; * Statistically significant; All p values are Bonferroni corrected

ANOVA testing comparing preoperative and last FU ABG from the whole cohort of patients demonstrated significant effects for time ($\:{F}_{(1,\:28)}=36.526,\:p<0.001,\:\:{\eta\:}_{p}^{2}=0.114$) and material ($\:{F}_{(7,\:28)}=7.245,\:p<0.001,\:\:{\eta\:}_{p}^{2}=0.152$). Upon stratifying the patients according to PORP and TORP techniques, an interaction between time and material was found in the TORP group only ($\:{F}_{(2,\:68)}=4.697,\:p=0.012,\:\:{\eta\:}_{p}^{2}=0.121$). There was no difference in pre- and postoperative ABG for the bony TORP technique; however, an improvement was observed for the semi-synthetic TORP, and an even more significant improvement was noted for the titanium TORP

**Fig. 1:**
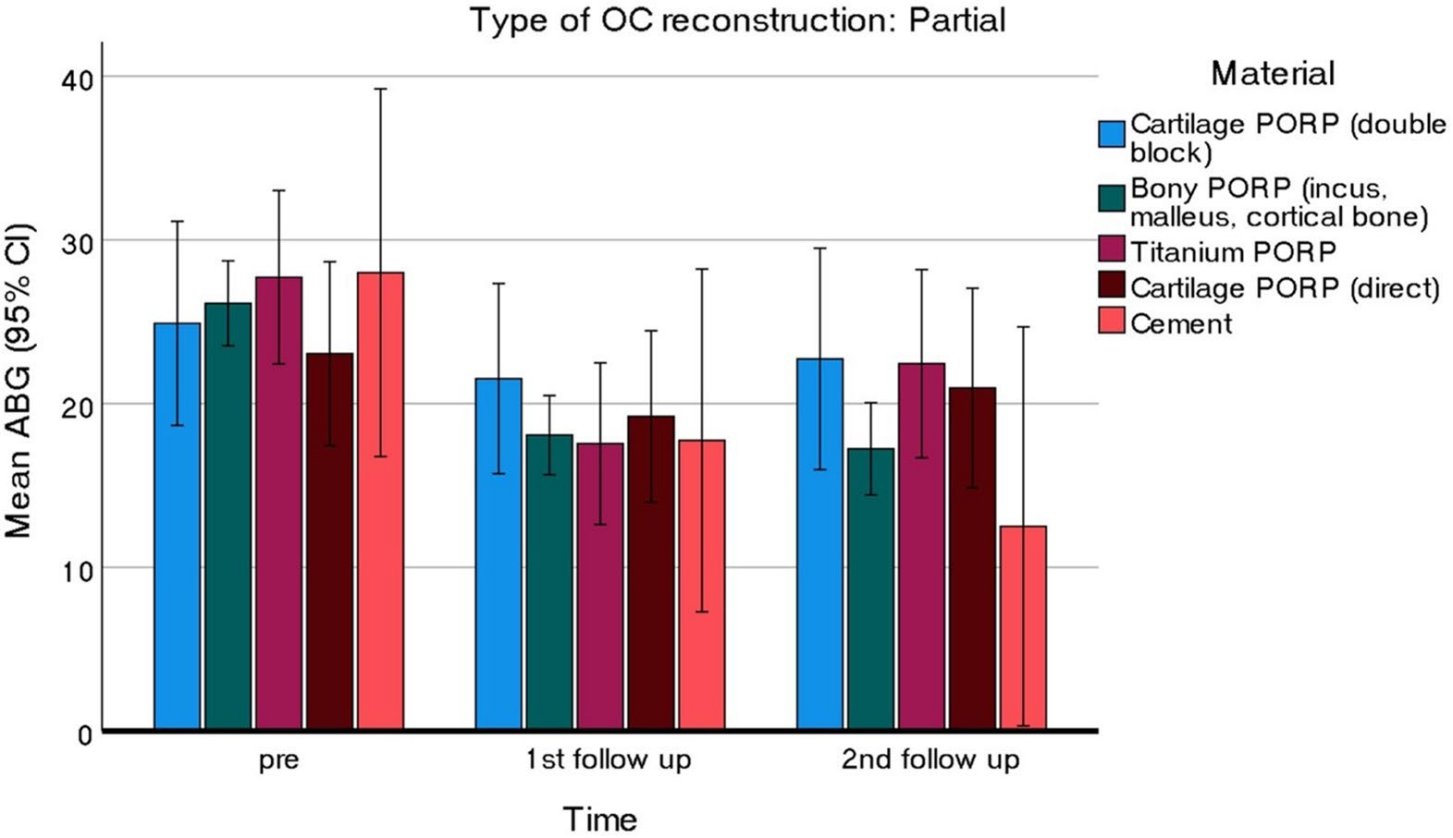
Hearing results from PORP group across two follow-up visits Abbreviations: ABG = Air-bone gap; OC = Ossicular chain; PORP = partial ossicular replacement prosthesis

**Fig. 2:**
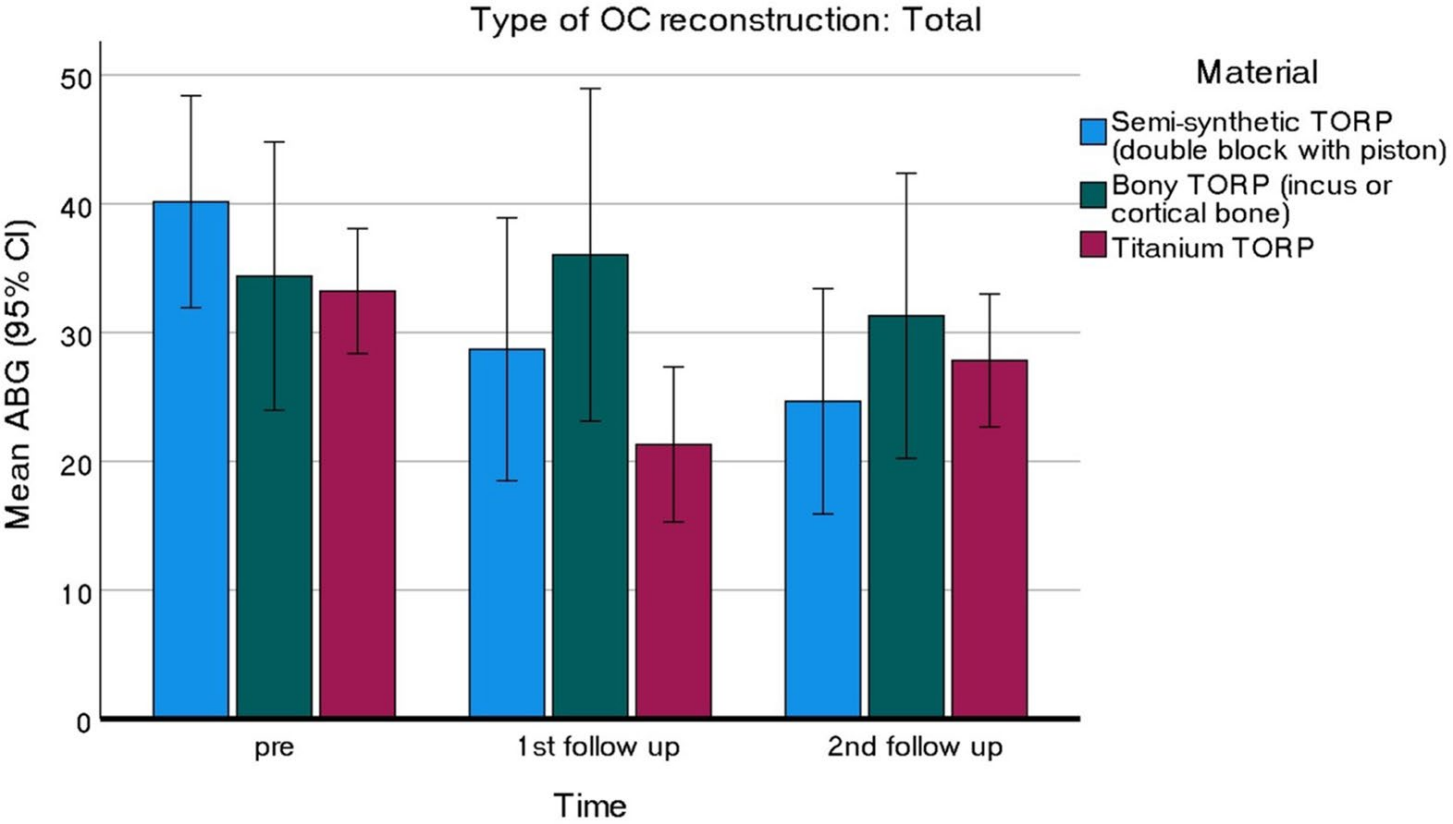
Hearing results from the TORP group across two follow-up visits Abbreviations: ABG = Air-bone gap; OC = Ossicular chain; TORP = total ossicular replacement prosthesis

Audiometric data from two FU visits (3 months and last FU) were obtained for a total of 162 cases, comprising 126 PORP and 36 TORP. As depicted in Fig. 1, both the cement PORP and bony PORP groups exhibited consistent improvements in ABG across the two FU visits. In contrast, the titanium based PORP and TORP initially demonstrated improvements in ABG but experienced a subsequent deterioration at the second FU (Figs. 1 and 2). A trend analysis over time revealed a significant quadratic trend for both titanium based PORP and TORP (PORP: $\:{F}_{(1,\:17)}=12.120,\:p=0.003,\:\:{\eta\:}_{p}^{2}=0.416$; TORP: $\:{F}_{(1,\:22)}=7.102,\:p=0.014,\:\:{\eta\:}_{p}^{2}=0.244$), while the linear trend was not significant in either case, indicating that ABG worsens rather than improves or remains the same from the first to the last FU. The cement reconstructions provided excellent results and remained stable over time

### Effects of surgical approaches on hearing results

**Fig. 3:**
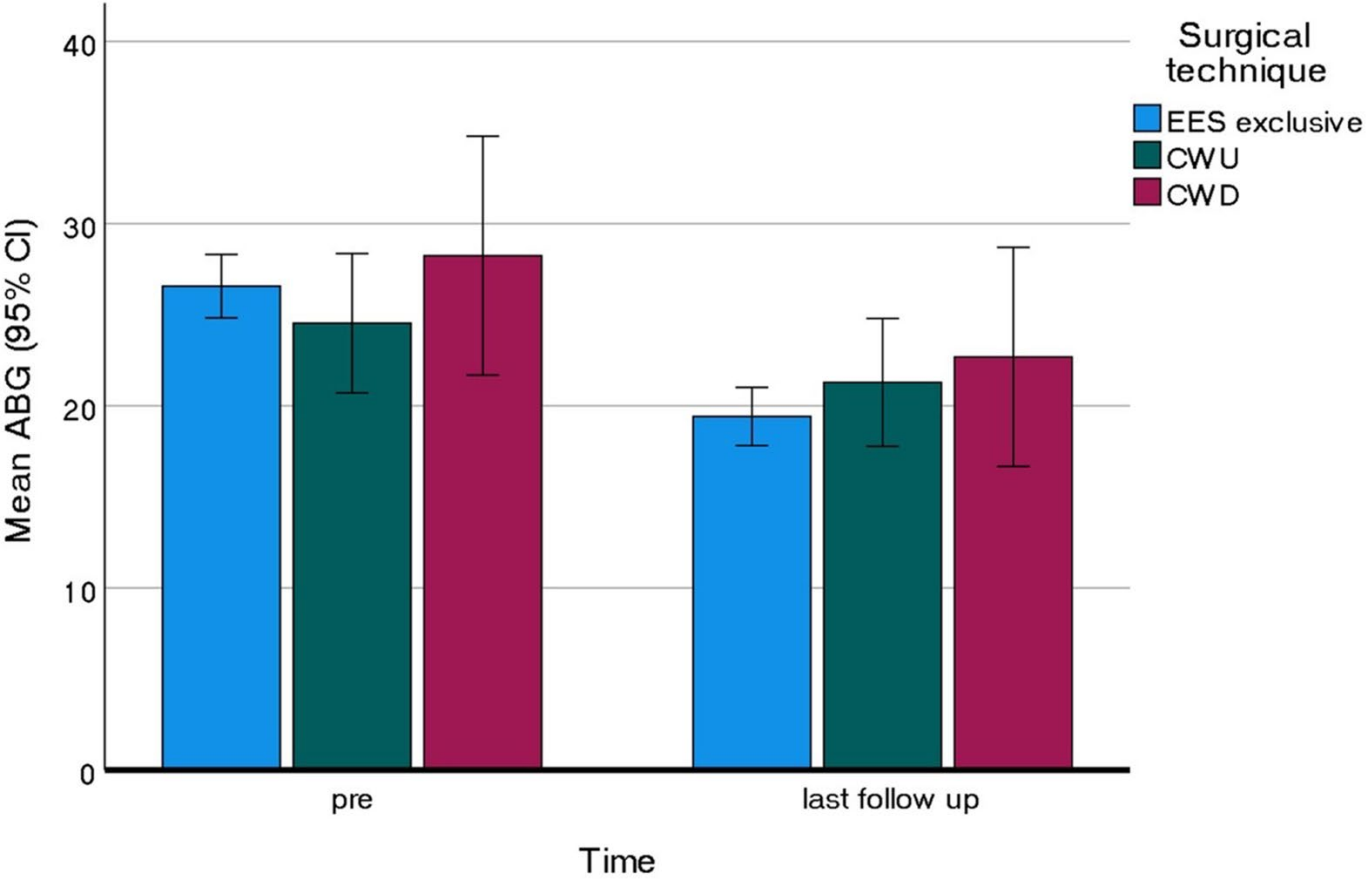
Comparison of the mean air-bone gap between preoperative and last follow-up Abbreviations: ABG = Air-bone gap; EES: endoscopic ear surgery; CWU: canal wall up; CWD: canal wall down

For a total of 271 cases, effects of surgical approach on hearing results were examined. A total of 59 cases with disease extension into the mastoid necessitated an additional CWU (*n* = 44) or CWD (*n* = 15) mastoidectomy, followed by endoscopic OPL. Figure 3 shows the improvement in hearing function at last FU in all surgical approaches. Only endo-OPL (without additional mastoidectomy) demonstrated a significant improvement in ABG ($\:{t}_{\left(211\right)}=6.861,\:p<0.001,\:\:{\eta\:}_{p}^{2}=0.182$). While the CWD group showed a comparable effect size ($\:{\eta\:}_{p}^{2}=0.176$), statistical significance was not reached likely due to the small sample size.

### Clinical follow-up

Among the 292 cases, clinical evaluation at last FU showed a graft success rate of 94.2%, with atelectasis of the TM observed in 29 cases (9.9%). In the cohort of patients receiving titanium PORP (*n* = 24), reported complications included one dislocation (4.2%) and two extrusions (8.4%). Among those treated with titanium TORP (*n* = 39), two dislocations occurred (5.2%), with no cases of extrusion.

## Discussion

This study reports audiological outcomes from a multicenter experience involving 292 patients who underwent endoscopic OPL for different pathologic conditions of the ossicular chain, primarily erosion from cholesteatoma. Overall, a significant audiological improvement was observed, suggesting the effectiveness of this endoscopic approach in improving the hearing function of patients affected by conductive or mixed hearing loss. Previous studies have confirmed the feasibility and success of OPL (conventionally defined as a reduction in postoperative ABG to levels below 20 decibels [3]) using a microscopic technique, with PORP typically exhibiting superior results compared to TORP procedures [22–26]. Our experience with the endoscopic technique, which is the largest reported in the literature so far, confirms these trends through a strict statistical analysis to adjust for potential errors, given the heterogeneity of the underlying diseases and techniques used. Notable variations in outcomes emerged within our cohort, based on specific PORP and TORP materials, as well as on surgical approaches.

Longitudinal audiometric data from two FU visits for a subset of patients (*n* = 162) provided insights into the stability of hearing improvements over time. Bony PORP, the most frequently used technique overall, was the only partial reconstruction technique associated with a significant improvement in ABG (post-operative ABG 16.42 dB, *p* < 0.001). The bony PORP and cement PORP groups demonstrated consistent ABG improvements across visits, suggesting these materials provide durable audiological outcomes. In contrast, titanium PORP and cartilage-based PORP, while showing improvement, did not achieve statistically significant postoperative gains. This observation is most probably due to the sample size in comparison to the bony group. Additionally, it is probable that titanium implants or cartilage-based PORP were used for cases with more ossicular chain destruction, since - whenever available - incus or malleus head interposition OPL were preferred for reconstruction.

Regarding bony PORP, several authors have reported significant hearing improvements using both incus and cortical bone grafts, with comparable outcomes when these materials are used for interposition OPL or myringostapedopexy. Chaudhary et al. documented a mean ABG closure of 22.05 dB with autologous incus and 25.02 dB with cortical bone. However no statistically significant difference in ABG closure was observed between the two materials [27]. As in our experience, the incus was more frequently utilized due to its availability in the surgical field, which eliminates the need for additional harvesting time.

A systematic review on PORP reconstructions with the microscopic technique, confirmed that the most utilized autologous technique for OPL in type III tympanoplasties is the sculpted incus autograft transposition [10]. According to this review, there is no significant difference in hearing outcomes between remodeled incus and synthetic PORPs, with mean postoperative ABG values of 18.97 dB and 16.27 dB, respectively. However, synthetic PORPs appear to have a higher likelihood of achieving ABG closure within 20 dB. In our series, the postoperative ABG in the bony PORP group was slightly lower, suggesting a possible role of the endoscopic technique in facilitating the proper positioning of the remodeled incus. This is in line with other endoscopic series, such as Soloperto et al. (postoperative ABG of the PORP group = 15.85 dB), and Das et al. (postoperative ABG in the endoscopic PORP group = 12.11 dB) at 6 months after surgery [20, 28]. On the other hand, in our experience titanium PORPs were used rarely (only 24 cases), limiting our ability to draw conclusions with external validity for this technique.

Previous studies on the use of cement in OPL report overall positive results, with ABG closure rates ranging from 60 to 94.4%. Baglam et al. reported the largest series to date involving 136 patients, achieving a postoperative ABG of less than 20 dB in 81.6% of cases after one year [29]. Similarly, Mohan et al. observed an average postoperative ABG improvement of 14 dB in a cohort of 25 patients who underwent PORP reconstruction with cement, with significant ABG closure in 76% of cases at 4 months postoperatively. Interestingly, the same authors emphasized key technical considerations for effective cement use in the middle ear, including meticulous bleeding control, detachment of mucosa from the ossicles, application of the cement within two minutes of mixing, and avoidance of contact with perilymph, dura mater, or neural structures [30]. The endoscopic approach may further facilitate adherence to these recommendations.

As commented by Celenk et al., bone cement OPL is the better choice for small defects between the incus remnant and the stapes head, because this method enables preservation of the ossicular chain, which is associated with better hearing results. For larger defects, the construction of a stable bridge with bone cement becomes challenging, and incus repositioning is generally recommended [31]. The limited number of cases in which cement was used in our series likely correlates with the advanced stage of ossicular chain erosion found in our population, with 70% of cases involving cholesteatoma.

One potential cause of failure with bony PORPs mentioned in the literature is bone resorption, though data are inconsistent on its prevalence. Histological studies of ossicular and cortical bone grafts reveal varying degrees of remodeling (1–83%), but reabsorption of the autologous bone graft has been identified only in cases involving recurrent cholesteatoma [10, 32–34]. On the contrary, Nikolaou et al. reported a 12.5% rate of resorption in the incus interposition group [35]. In our series, no cases of bone resorption are documented, with stable audiological results over time.

Conversely, weak long-term results with synthetic PORPs and TORPs may be attributed to extrusion or dislocation over time, which remain notable challenges in ossicular reconstruction despite their biocompatibility, lightweight nature, and ease of placement [36–38]. Such complications may be independent on the type of approach (microscopic vs. endoscopic), as well as the reconstruction technique (TORP vs. PORP). In our series, titanium-based TORP showed initial improvement, with an improved mean postoperative ABG, followed by a decline at the second FU. The rate of documented dislocation and extrusion in our cohort was 5.2% for the TORP group and 12.6% for the PORP group, with a mean postoperative ABG of 24.44 and 22.33dB respectively. In the literature, titanium prostheses are associated with extrusion rates, ranging from 0.9 to 16.3% (mean postoperative ABG ranging from 18.1dB to 24.6dB), with most studies reporting rates closer to the lower end [37]. This is often attributed to factors such as off-axis placement, persistent middle ear disease or Eustachian tube dysfunction. Meta-analyses indicate that TORPs have significantly higher long-term extrusion rates compared to PORPs, likely due to reduced stability and the severity of the underlying disease [39]. In our experience the rate was higher within the PORP group and overall low. It should be underlined that in our cohort not all patients with poor or decreased hearing over time after OPL were surgically revised, thus the actual percentage of dislocation events may be underestimated.

While extrusion is the most common complication, prosthesis displacement into other areas of the middle or inner ear can also occur. Medial displacement into the vestibule may result in fistula-like symptoms, such as vertigo and hearing loss, as in cases of TORP medialization. However, TORP medialization is a rare occurrence and is likely caused by prostheses that are excessively long, making it more of a theoretical concern, as discussed by Polanik and Remenschneider [40]. Patients with active inflammation of the middle ear at time of surgery or mastoid obliteration, whether prior or simultaneous to TORP placement, may be at increased risk of prosthesis migration due to anatomical distortion. To minimize failure rates, literature has suggested to stage the tympanic membrane repair and OPL to stabilize the middle ear before prosthesis placement [10, 39]. In regards of the drawbacks of the staged reconstruction and the encouraging results of our cohort, primary OPL can be considered a valid option.

Interestingly, among the TORP options in our cohort, synthetic prostheses provided better outcomes than autologous ones. Specifically, titanium TORPs achieved a lower mean postoperative ABG of 24.44dB compared to semi-synthetic TORPs with a meand postoperative ABG of 27.05dB (*p* > 0.05), while bony TORP showed the poorest outcomes, with no significant ABG gain. This could be explained by certain design features of these synthetic prostheses: the open-head design facilitates manipulation and provides an unobstructed view of the footplate during placement, ensuring proper positioning more than with larger cartilage or bony blocks. Based on these results, it may be hypothesized that the improved visualization provided by the endoscopic technique is not sufficient to guarantee optimal positioning of bulky prostheses.

The literature on outcomes with synthetic versus autologous prosthesis remains inconclusive, both in terms of audiological outcomes and stability over time. Similarly to our experience, Kong et al. found that titanium TORP had more favorable hearing gain than autologous grafts [41]. Van Holst et al. also reported favorable functional outcomes with titanium TORP, with these results maintained over an extended FU period [42]. A systematic review by Kortebein further indicated that titanium prostheses reliably improve hearing outcomes even after 12 months, with ABG improvements of 12 dB for PORPs and 17 dB for TORPs [11]. Conversely, other studies found that non-titanium TORP yielded better outcomes when compared with titanium, suggesting tissue integration as a possible mechanism facilitating the transmission of sound in such patients [43–45]. A recent meta-analysis showed that titanium prostheses did not demonstrate significant superiority over non-titanium prostheses in terms of effectiveness and stability [12], while Hillman and Shelton showed worse results with titanium prostheses versus Plastipore prostheses [43]. These contradictory findings suggest that factors beyond material type, such as middle ear ventilation, mucosal conditions, as well as surgeon’s experience, may significantly influence long-term outcomes. Unfortunately, such variables are rarely reported in these studies, probably due to a lack of universally accepted scoring systems or objective tools to stratify these intraoperative features. In this regard, our study also suffers from this limitation due to its retrospective nature.

While several studies have compared hearing results between CWU and CWD mastoidectomies, ours is among the few which investigate how mastoid preservation could influence postoperative hearing after OPL. In our cohort, exclusive endoscopic procedures were significantly associated with overall improved postoperative hearing, while additional CWU and CWD mastoidectomy did not correlate with hearing improvement. In line with our findings, Desaulty et al. demonstrated that patients have better results without mastoidectomy than those undergone concomitant mastoidectomy [45]. 

This phenomenon may be interpreted in light of the mastoid preservation theory, which suggests that a well-functioning, pathology-free mastoid positively contributes to hearing function [46]. Moreover, the lesser extend of disease in the mastoid-sparing surgical techniques may also contribute to an improved postoperative hearing result. Despite these encouraging findings, larger studies are needed to corroborate these results. Furthermore, this evidence could be useful during preoperative counseling: for this reason, candidates to ear surgery with limited disease manageable through an exclusive endoscopic approach may be informed that this technique allows for effective hearing rehabilitation through endo-OPL.

### Limitations

We acknowledge the limitations of the present study mainly due to its retrospective nature and its inherent challenges, for example, the possible presence of confounding variables, the vulnerability to the development of a selection bias and the fact that we cannot determine causation, only association. Besides, our study has an asymmetry in terms of group size in terms of use of different OPL materials and techniques.

## Conclusion

This multicenter study supports the efficacy of endo-OPL as a valuable technique to improve hearing outcomes in patients with conductive or mixed hearing loss. While bony or cement PORP showed sustained audiological benefits across FU period, titanium prostheses, though initially effective, demonstrated potential long-term stability issues. Additionally, this study underscores the potential of mastoid preservation, with exclusive endoscopic procedures providing better postoperative hearing outcomes than those undergone to additional mastoidectomy.
